# Interaction between Various Apple Procyanidin and Staphylococcal Enterotoxin A and Their Inhibitory Effects on Toxin Activity

**DOI:** 10.3390/toxins9080243

**Published:** 2017-08-07

**Authors:** Yuko Shimamura, Chikako Hirai, Yuka Sugiyama, Mio Utsumi, Akio Yanagida, Masatsune Murata, Norio Ohashi, Shuichi Masuda

**Affiliations:** 1 School of Food and Nutritional Sciences, University of Shizuoka, 52-1 Yada, Suruga-ku, Shizuoka 422-8526, Japan; shimamura@u-shizuoka-ken.ac.jp (Y.S.); gp1848@u-shizuoka-ken.ac.jp (C.H.); gp1534@u-shizuoka-ken.ac.jp (Y.S.); s16204@u-shizuoka-ken.ac.jp (M.U.); ohashi@u-shizuoka-ken.ac.jp (N.O.); 2 School of Pharmacy, Tokyo University of Pharmacy and Life Sciences, 1432-1 Horinouchi, Hachioji, Tokyo 192-0392, Japan; yanagida@toyaku.ac.jp; 3 Department of Nutrition and Food Science, Ochanomizu University, 2-1-1 Otsuka, Bunkyo-ku, Tokyo 112-8610, Japan; murata.masatsune@ocha.ac.jp

**Keywords:** staphylococcal enterotoxin A, *Staphylococcus aureus*, procyanidin, polymerization, apple

## Abstract

In this study, we investigated the interaction between apple polyphenols (AP; mainly consisting of procyanidin (PC) from an apple) and staphylococcal enterotoxin A (SEA), and the inhibitory effects of AP on SEA activity. According to the degree of polymerization, in particularly highly polymerized PC (more than pentamer) strongly interacted with SEA. The binding affinity of AP with SEA molecules was determined using Biacore analysis. AP reacted with SEA immobilized on a Biacore sensor chip. After treatment with pepsin and pancreatin, to examine the changes of binding affinity of AP in intragastric conditions, AP maintained interaction with SEA. We examined whether AP inhibits the proliferation and interferon-γ (IFN-γ) production induced by SEA in mouse spleen cells. AP strongly inactivated the proliferation and IFN-γ production induced by SEA. These results suggest that AP, which has a higher degree of polymerization, inactivates stronger biological activity of SEA through interaction with SEA. Our studies are the first to demonstrate the relationship between the degree of polymerization of AP and the inhibitory effects on SEA activities.

## 1. Introduction

*Staphylococcus aureus* (*S. aureus*) belongs to the normal flora of humans. However, it is thought that virulent strains may infect skin or cause staphylococcal food poisoning. Staphylococcal food poisoning is due to the intake of staphylococcal enterotoxins (SEs) [1]. *S. aureus* produces groups of 11 SEs and 11 SE-like toxins [2]. Among the SEs, staphylococcal enterotoxin A (SEA) is the most common cause of staphylococcal food poisoning worldwide [3]. Therefore, it is necessary to control SEA-mediated food poisoning.

It was reported that apple polyphenols (AP) inhibited SEA-induced biological activity [4]. Apples contain several phenolic substances (i.e., chlorogenic acid, (+)-catechin, epicatechin, phloridzin, rutin, and oligomeric procyanidins (PC)) [5]. The PC in apples is mainly composed of various polymerized catechins. Ohnishi-Kameyama et al. reported that concentrated tannins, extracted from unripe apple juice, contained polymerized catechins including pentadecamers [6]. Shoji et al. (2006) demonstrated that apple procyanidins were able to be fractionated according to the degree of polymerization [7]. The inhibitory effects of polyphenols on SEA toxin activity may depend on polyphenol structures and particularly their degree of polymerization. However, the inhibitory effect of them on toxin activity has not yet been examined through differences in polyphenolic degrees of polymerization.

In the present study, we examined the interaction between AP and SEA, and investigated the interaction strength of fractionated PC according to the degree of polymerization at concentrations without inhibiting growth of *S. aureus*. In addition, we inquired into the interaction of AP and SEA within the digestive tract by using the intragastric enteral model in vitro. With the interaction of SEA and AP, we further examined whether AP inhibited the proliferation and interferon-γ (IFN-γ) production induced by SEA in mouse spleen cells.

## 2. Results

### 2.1. Fractionation of AP Mixture

**Figure 1:**
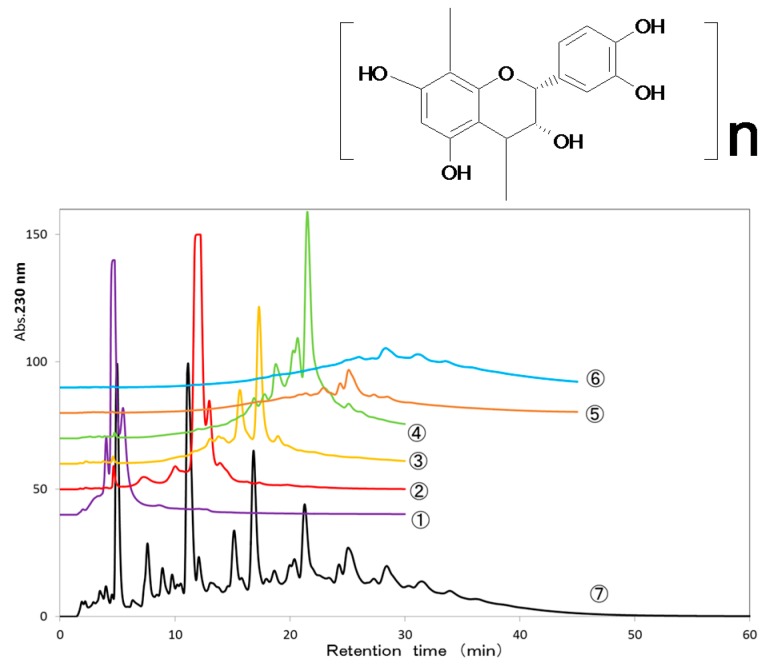
Analytical HPLC chromatograms of the oligomeric procyanidin (PC) constituents in crude apple polyphenols (AP) mixture (No. 7) and those in preparative HPLC fractions (No. 1–6) obtained from the AP mixture. Both conditions of the preparative and analytical HPLCs are described in the Section 4.2. The oligomeric PC constituents in the fractions 1–6 are as follows; No. 1 (i.e., fr.1): monomer; No. 2: dimer; No. 3: trimer; No. 4: tetramer; No. 5: pentamer; No. 6: greater than pentamer.

The samples containing proanthocyanidin from apples were applied to a TSKgel Amide-80 column (21.5 × 300 mm I.D., 10 μm, Tosoh Corporation, Tokyo, Japan) with acetonitrile containing decreasing proportions of acetonitrile (90–50%) to yield a monomer (82.9 mg; molecular weight (MW) 288 Da), dimer (22.8 mg; MW 576 Da), trimer (24.9 mg; MW 864 Da), tetramer (19.8 mg; MW 1152 Da), pentamer (13.3 mg; MW 1440 Da) and greater than pentamer (15.8 mg). The molecular weight of the more than pentamer fraction was regarded as 1728 Da (Figure 1).

### 2.2. Effect of AP and PC Fractions on S. aureus Growth

To examine the effect of AP and PC fractions on *S. aureus* growth, MIC was determined by broth microdilution assays. When sample concentrations were less than 5 mg/mL (AP) and 3 mM (each PC fraction), they had no effect on *S. aureus* growth. All following assays were performed at non-growth inhibitory concentrations (less than MIC values).

### 2.3. Interaction of PC Fractions with Cultured SEA-Producing Strain and Purified SEA

**Figure 2:**
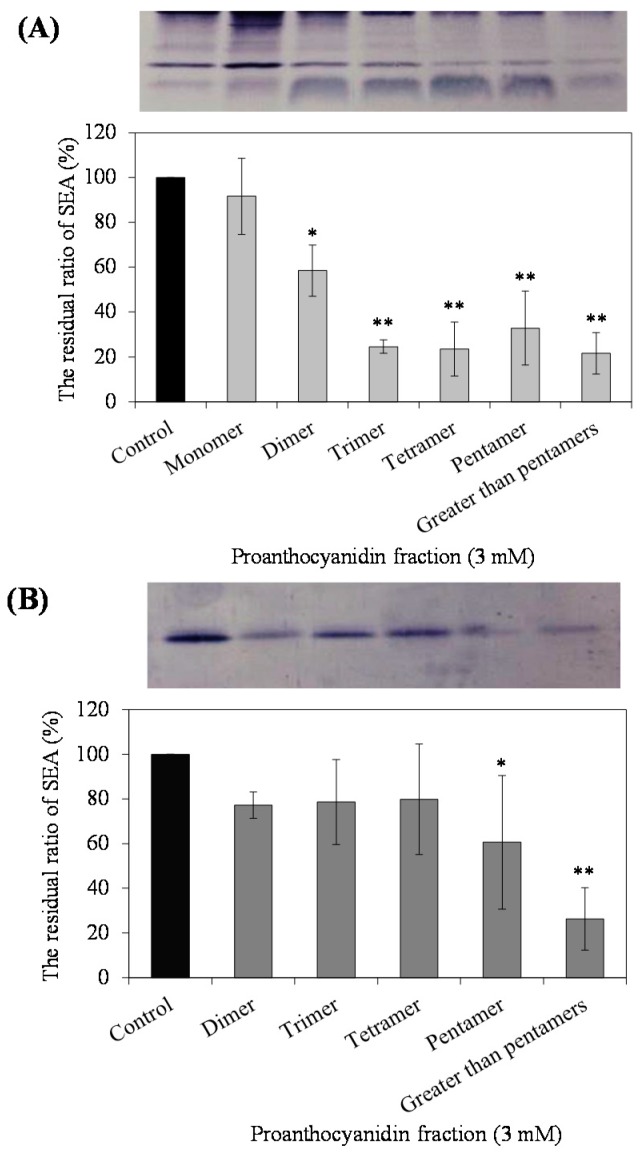
Interaction between procyanidin (PC) fractions and cultured staphylococcal enterotoxin A (SEA) producing strain or purified SEA. (**A**) Interaction between PC fractions and cultured SEA-producing strains. *Staphylococcus aureus* C-29 was cultured with six PC fractions in BHI broth for 24 h at 37 °C; (**B**) Interaction between purified SEA and five PC fractions (di-, tri-, tetra-, penta-, and greater than pentamer) of treated or untreated SEA were incubated for 24 h at 37 °C. Then test samples of treated or untreated SEA were separated by SDS-PAGE under reducing conditions and analyzed by immunoblotting with anti-SEA antibodies. * represents *p* < 0.05 compared to the control, ** represents *p* < 0.01 compared to the control. Values represent the mean ± SD for three independent experiments.

**Figure 3:**
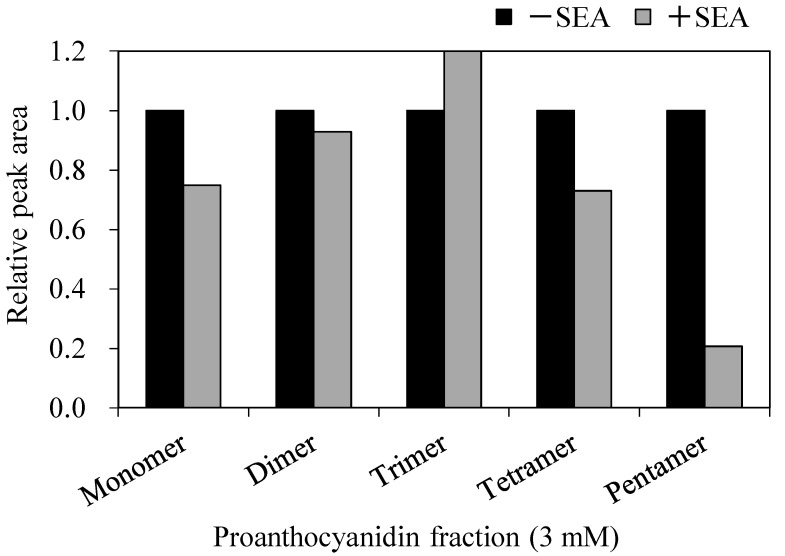
Relative peak area of procyanidin (PC) with or without staphylococcal enterotoxin A (SEA) on the HPLC chromatograms. Each PC fraction (final concentration of 1.5 mM) and purified SEA (final concentration of 100 ng/mL) were mixed and incubated at 37 °C for 24 h. Each fraction was analyzed by HPLC (column, Agilent Zorbax SB-C18 (4.6 × 150 mm I.D., 1.8 μm, Agilent Technologies, Palo Alto, CA, USA); mobile phase 0.5% trifluoroacetic acid (TFA): acetonitrile (87:13); flow rate of 0.6 mL/min). HPLC analysis was repeated twice.

It was examined which PCs included in the AP showed strong interaction with SEA. The interaction of each PC fraction with the SEA produced by *S. aureus* C-29, which is an SEA-producing strain, was examined using Western blot analysis. We conducted experiments with almost equal number of *S. aureus* C-29 after treatment with each PC fraction (Control; 4.15 ± 0.10, monomer; 4.22 ± 0.05; dimer; 4.18 ± 0.07, trimer; 4.27 ± 0.04, tetramer; 4.12 ± 0.02, pentamer; 4.11 ± 0.16, greater than pentamer; 4.71 ± 0.11 logCFU/mL). As a result, fractions at 3.0 mM PC, except for monomers, significantly decreased the band intensity of SEA protein (*p* < 0.05) (Figure 2A). Since it is possible that these five PC fractions (di-, tri-, tetra-, penta-, and greater than pentamer), except for monomers, directly reacted with the SEA, we examined whether they could interact with purified SEA itself. As a result, highly polymerized PC (pentamer, and greater-than-pentamer fractions) showed interaction with SEA (*p* < 0.05) (Figure 2B). In contrast, SEA was not precipitated by treatment with low-polymerized PC (dimer to tetramer fractions). These results may mean that most of the non-reacted SEA remained in the supernatant by separating the mixture of dimer to tetramer fractions and SEA (Figure 2B). The PC in the supernatant after reaction with SEA was measured using HPLC. Most of the low-polymerized PCs (di-, tri-, tetramer) were found in the supernatant by separating the mixture of PC and SEA. However, highly polymerized PCs (pentamer) remained only in approximately 20% of the supernatant (Figure 3). From these results, highly polymerized PC (penta-, and greater-than-pentamer fractions) interacted with SEA stronger than other PC fractions contained in AP. Therefore, it is suggested that the interaction of AP with SEA might be contributed to by pentamer and greater-than-pentamer fractions.

### 2.4. Estimation of Binding Activity of AP to SEA Using Biacore Analysis

**Figure 4:**
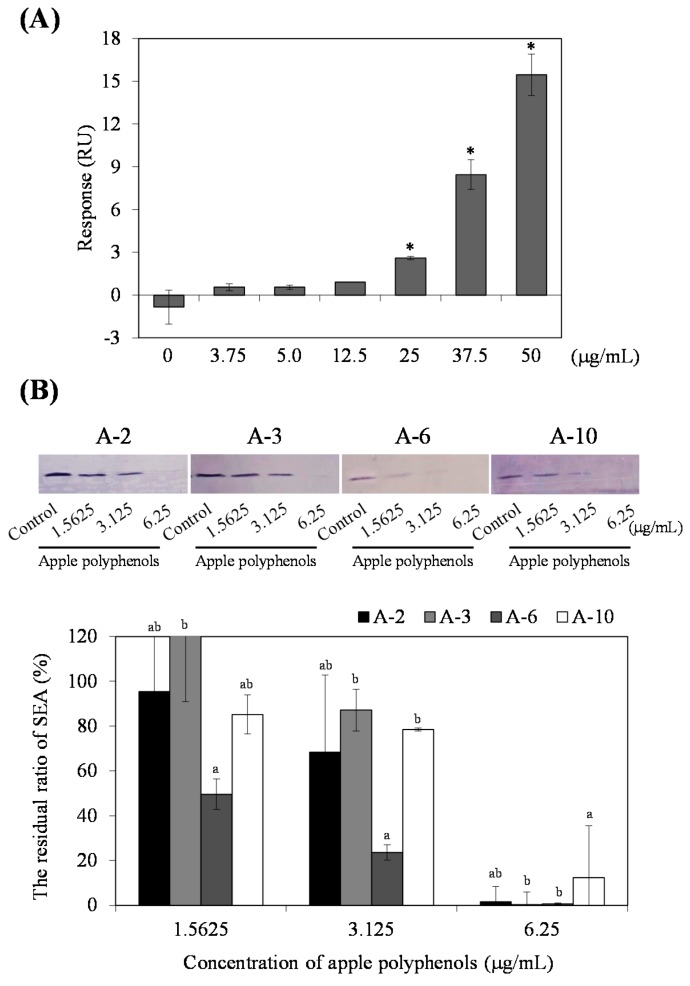
Reaction of staphylococcal enterotoxin A (SEA) and apple polyphenols (AP). (**A**) Biacore analysis of the binding of AP to SEA. The affinity of AP was examined by surface resonance on a Biacore 2000 biosensor instrument, with SEA immobilized on the flow cell of the sensor chip CM5. Binding responses are represented in resonance units (RU). A running buffer was used as a negative control (0 μg/mL). * represents *p* < 0.05 compared to the control; (**B**) The interaction of AP on SEA active sites. Each sample was mixed with SEA (4.5 μg/mL) in 50 μL of MilliQ water and incubated at 37 °C for 24 h. Following centrifugation, the supernatant was applied to SDS-PAGE and visualized by Western blot analysis. MilliQ water was performed as a positive control. The residual ratio of SEA protein were determined by Western blot analysis and quantified using ImageJ software (National Institutes of Health, Bethesda, MD, USA). There is a significant difference between different alphabets (Tukey–Kramer test, *p* < 0.05). Values represent the mean ± SD for three independent experiments.

The molecular basis of the binding of AP to SEA was investigated by using surface plasmon resonance (SPR)-based binding techniques. The affinity of AP (0, 3.75, 5.0, 12.5, 25, 37.5 and 50 μg/mL) to SEA was examined by surface resonance on a Biacore 2000 biosensor instrument, with SEA immobilized on the flow cell of the sensor chip CM5. The value of 966.5 RU was obtained as the maximum response of SEA immobilization. A difference from blank (AP 0 μg/mL) was significantly increased at concentrations of more than 25 µg/mL of AP. This reaction was achieved with 25 µg/mL of AP injected over the chip at 20 µL/min for three minutes (Figure 4A).

### 2.5. Interaction of AP with SEA Active Sites

Among the PC fractions included in the AP, it was suggested that highly polymerized PC (pentamer and greater-than-pentamer fractions) strongly interact with SEA. Therefore, focusing on the SEA active sites, it was examined which SEA active sites interacted with AP. The interaction of AP with toxin active sites was analyzed using four different prepared synthetic peptides equivalent to the SEA toxin active site and rabbit antibodies with their corresponding peptides (A-2, A-3, A-6 and A-10). Treatment with AP showed an interaction with SEA active sites in a dose-dependent manner. Especially the strong inhibitory effect of AP on the interaction of an anti-peptide antibody with A-6 (corresponding to amino acid residues 81–96) was found (Figure 4B). These results suggest that highly polymerized PC included in AP interacts with the A-6 region of SEA.

### 2.6. Effect of pH, Pepsin and Pancreatin Treatment on Interaction of AP with SEA

**Figure 5:**
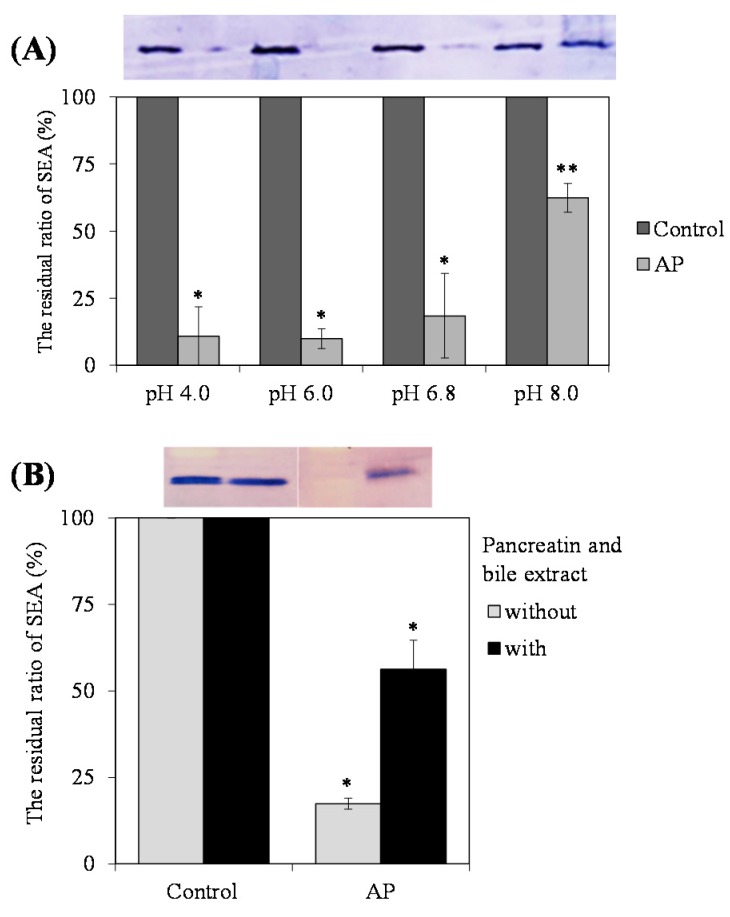
The interaction of apple polyphenols (AP) to staphylococcal enterotoxin A (SEA) in various conditions. (**A**) The interaction of AP to SEA in various pH conditions. SEA (4.5 μg/mL) and AP (2.5 mg/mL) in 100 μL of Mcilvaine buffer (pH 4.0–8.0) were incubated at 37 °C for 24 h. Following centrifugation, the supernatant was applied to SDS-PAGE and visualized by Western blot analysis. MilliQ water was used as a positive control. * represents *p* < 0.05 compared to the control, ** represents *p* < 0.01 compared to the control; (**B**) The interaction of AP to SEA in gastrointestinal model solution. AP (2.5 mg/mL) and SEA mixture was adjusted to a pH of 2.4 using Mcilvaine buffer, followed by the addition of pepsin from porcine gastric mucosa (final concentration of 0.32%). After three hours, the solution was adjusted from a pH of five to a pH of six to inactivate pepsin, followed by the addition of pancreatin from porcine pancreas (final concentration 0.25%) and bile powder (final concentration 0.4%). After 24 h, the reaction mixture was used for SDS-PAGE and Western blot analysis. MilliQ water was used as a positive control. * represents *p* < 0.05 compared to the control. Values represent the mean ± SD for three independent experiments.

It was confirmed whether the AP including highly polymerized PC interacted even in the various pH and gastrointestinal model solutions. At first, we examined the effect of pH (Mcilvaine buffer; pH 4.0, 6.0, 6.8 and 8.0) on the interaction of AP with SEA. After treatment at acidic and alkaline conditions (pH 4.0–8.0), AP still maintained the interaction with SEA (Figure 5A). Although the interaction of AP with SEA was maintained at all pH levels, its optimal interaction appeared in the range of pH 6.0 and below. In addition, after in vitro treatment with pepsin (three hours), followed by pancreatin (24 h), the interactive activity of AP with SEA was maintained (Figure 5B).

### 2.7. Inhibitory Effect of AP and PC on Toxic Activity and IFN-γ Production Induced by SEA

**Figure 6:**
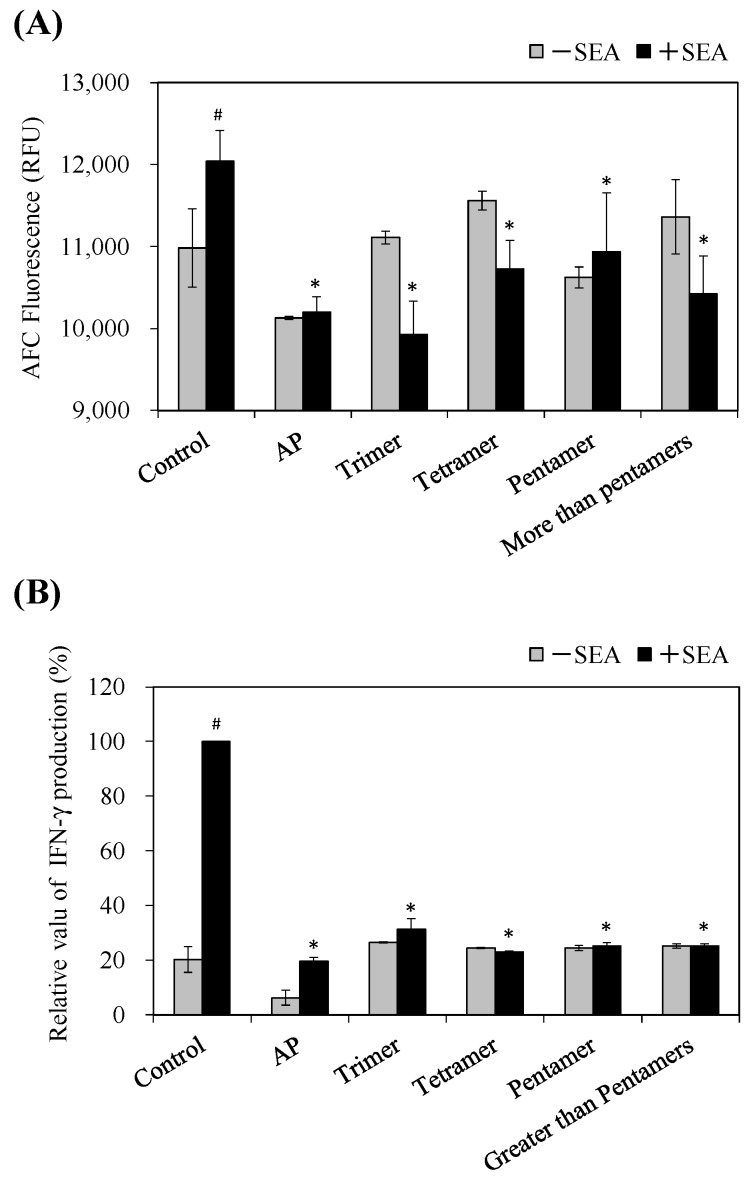
Effect of apple polyphenols (AP) on toxin activity and cytokine production induced by staphylococcal enteroroxin A (SEA). (**A**) Inhibitory effects of procyanidin (PC) fractions on SEA toxin activity. SEA was exposed to PC fractions or the control (media) and then incubated for 48 h with splenocyte cells. GF-AFC cleavage by splenocyte proliferation was measured by fluorescent AFC, which is quantified at an excitation wavelength of 400 nm and an emission wavelength of 505 nm. Distilled water was used as a control (Cont.). # represents *p* < 0.05 compared to the SEA (−) control, * represents *p* < 0.05 compared to the SEA (+) control; (**B**) Inhibitory effects of procyanidin (PC) fractions on SEA-induced IFN-γ production. AP (0.1 μg/mL) or each PC fraction (0.1 mM) with or without SEA (100 ng/mL) was incubated for 72 h with splenocyte cells, and the IFN-γ production was determined by ELISA. IFN-γ production of SEA alone (SEA (+)) was taken as 100%. SEA (100 ng/mL) was used as a positive control. # represents *p* < 0.05 compared to the SEA (−) control, * represents *p* < 0.05 compared to the SEA (+) control. Values represent the mean ± SD for three independent experiments.

The relationship between the interaction of AP or PC with SEA protein and the inhibition of biological activity was examined. At first, we examined whether highly polymerized PC (pentamer and greater-than-pentamer) fractions and low polymerized PC (tri-, and tetramer) fractions showed interaction with SEA, or inhibited SEA activity. These PC fractions were incubated with and without SEA (100 ng/mL) in a Russ-10 medium with mouse spleen cells from C57BL/6J female mice for 48 h. The viable cell counts were not changed in the spleen cells treated only with AP or PC fractions at a final concentration of 0.1 mg/mL or 0.1 mM, respectively. The cell proliferation of spleen cells induced by SEA (100 ng/mL) was examined using the GF-AFC assays. As shown in Figure 6A, AP or PC fractions inhibited cell proliferation induced by SEA (*p* < 0.05). There is no significant difference among samples under conditions without SEA (-SEA). It has become clear that SEA induces the release of cytokines after the proliferation of a large number of T cells. In particular, it appears that AP alone reduced the cell response relative to the more refined oligomeric forms. Since AP including each PC fraction inhibited SEA-induced cell proliferation, we also examined whether AP or PC fractions can inhibit SEA-induced IFN-γ production. Treatment with AP (fractions at a final concentration of 0.1 μg/mL) or PC fractions (0.1 mM) significantly showed the inhibitory effect on SEA-induced IFN-γ production (*p* < 0.05) (Figure 6B).

### 2.8. Real-Time RT-PCR

**Figure 7:**
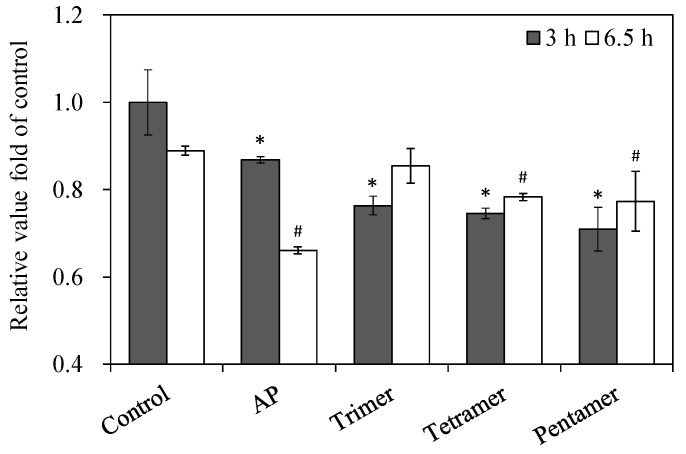
Relative gene expression of sea in *Staphylococcus aureus* C-29 after incubation with apple polyphenols (AP) and procyanidin (PC) fraction. Samples for RNA extraction were taken at two different times (three hour and 6.5 h old cultures). * represents *p* < 0.05 compared to the control of the three hour incubation. # represents *p* < 0.05 compared to the control of the 6.5 h incubation. Values represent the mean ± SD for three independent experiments.

Based on findings that suggested that low polymerized proanthocyanidins inhibit the production of SEA, we used a real-time RT-PCR assay to further investigate the relative expression levels of sea toxin-encoding genes after treatment with AP, low polymerized PC (tri-, tetra-, and pentamer) fractions. *S. aureus* C-29 expressed SEA genes from the early-stationary growth phase (three-hour-old cultures) to the logarithmic growth phase (6.5-hour-old culture) [8]. As shown in Figure 7, all samples significantly inhibited the transcription of SEA in *S. aureus* C-29 at the early-stationary growth phase and the logarithmic growth phase (*p* < 0.05).

## 3. Discussion

In this study, we examined the interaction between AP and SEA at concentrations not inhibiting growth of *S. aureus*. Our results for the MIC of each proanthocyanidin fraction against *S. aureus* (>5.0 mM) were higher than previous reports [9,10]. It seems that such differences could be attributed, on occasion, to different assays being used. It is reported that AP inhibited SEA-induced biological activity [4]. However, it is unknown whether AP interacts with SEA. Tenore et al. demonstrated that apple polyphenolic compounds bind to plasma proteins (human serum albumin, high density lipoprotein, low density lipoprotein) [11]. Therefore, it is likely that AP binds to SEA proteins as well as plasma proteins. To examine the interaction effects of each PC that are major components in AP mixtures, AP mixtures were separated to six PC fractions (mono-, di-, tri-, tetra-, penta-, and greater than pentamer). At first, the changes of interaction between SEA and PC, according to the degree of polymerization at concentrations not inhibiting the growth of *S. aureus*, was examined.

The effect of each PC fraction on the SEA-producing strain of *S. aureus* C-29, and purified SEA itself, was examined using Western blot analysis. As a result, while for PC fractions, except for monomers, it was suggested that there was an interaction between the PC fractions and the crude SEA expressed by *S. aureus* C-29 (*p* < 0.05) (Figure 2A), highly polymerized PCs (pentamer and greater-than-pentamer fractions) interacted with SEA (*p* < 0.05) (Figure 2B). In HPLC analysis, most of the low polymerized PC (di-, tri-, tetramer) was found in the supernatant after centrifugation of the reaction mixture, whereas highly polymerized PC (pentamer) remained only approximately 20% in the supernatant (Figure 3). These results suggest that proanthocyanidin fractions, except for the monomer, inhibit the production of SEA from *S. aureus* C-29, and highly polymerized PC reacts directly with SEA itself. These affinity interactions of procyanidins and SEA might depend on their degree of polymerization. It was reported that the interaction of PC and protein increased according to the degree of polymerization [12]. Our results for the interaction of PC with SEA protein was similar to previous reports. The polyphenol that interacts strongly with the protein is small enough to pass through the protein molecules and must be large enough to crosslink the peptide chains [13]. Additionally, in our previous study, the molecular weight of the polyphenols that weakly interacted was more than 1000, whereas the molecular weight of the polyphenols that strongly interacted was 1000 or less [14]. In the dimer to tetramer group, it was suggested that the interaction was weak when the molecular weight of polyphenol was less than or equal to 1000. In Biacore analysis, AP was immediately reactive with SEA immobilized on a chip in a dose dependent manner (Figure 4A). Binding to SEA was achieved with 25 µg/mL AP injected over the chip at 20 µL/min for three minutes. These results suggest that AP was immediately bound to SEA molecules in a short time.

The interaction affinities of AP with SEA toxin active sites (emetic and superantigenic activities) were analyzed using four different synthetic peptides and rabbit antibodies with their corresponding peptides (A-2, 21–40; A-3, 35–50; A-6, 81–100; or A-10, 161–180) [15]. Amino acid residues 21–50 and 81–100 of SEA molecule are necessary to superantigenic and emetic activities in house musk shrews [14]. Treatment with AP resulted in a dose-dependent interaction of SEA active sites. In particular, the strong inhibitory effect of AP was found in the interaction of an anti-peptide antibody with A-6 (corresponding to amino acid residues 81–96) (Figure 4B). Therefore, it is possible that AP might inhibit SEA-induced toxin activity.

Our studies suggest that AP interacts with SEA toxin active sites. Because SEA is resistant to low pH conditions and proteolytic enzymes, their toxic activity remains in the digestive tract after ingestion [16]. Therefore, we confirmed the effects of pH and digestive tract model in vitro on the interaction of AP with SEA. After treatment at acidic and alkaline conditions (pH 4.0–8.0), and in vitro digestion with pepsin followed by pancreatin, AP still interacted with SEA (Figure 5A). These results suggest that interaction between AP and SEA may be maintained in the digestive tract in vivo and the toxic activity of SEA is kept inactivated. However, the optimal interaction between AP and SEA appears to be at pH 6.0 and below. It was reported that the pH increases in the small intestine from pH 6.0 to about pH 7.4 in the terminal ileum [17]. Since these interactions have not yet been demonstrated in vivo, it will be necessary to examine whether the interactions are maintained in vivo using experimental animals in the future. Although the interactive activity of AP with SEA was maintained after treatment with pancreatin, the interaction between SEA and AP became weaker. Polyphenols such as tannins were reported to have binding affinity to the lipase contained in pancreatin [18]. It has also been reported that proanthocyanidins bind to the amylase contained in pancreatin [19]. It was suggested that the interaction between SEA and AP was weakened due to the binding of pancreatin enzyme and AP. Further work on the interaction between pancreatin enzyme and AP is required to confirm this possibility.

SEAs are superantigenic toxins which form the bridge between antigen presenting cells (APCs) and T cells. The cross-linkage formation induces T-cell proliferation and produces T-cell-activating cytokines such as IFN-γ [20,21]. Rasooly et al. (2010) reported that apple juice inhibited SEA induced T-cell proliferation by blocking the connection between AP and T cells [4]. Then we examined whether AP and PC fractions inhibit superantigenic activity using spleen cell proliferation and IFN-γ production induced by SEA in murine spleen cells from C57BL/6J mice as an evaluation system. As a result, AP and PC fractions (tri-, tetra-, penta-, and greater-than-pentamer) inhibited SEA-induced spleen cell proliferation (Figure 6A). Furthermore, AP and PC fractions inactivated IFN-γ production in spleen cells induced by SEA (Figure 6B). These results suggest that the highly polymerized PCs in apple extract inhibit toxic activity by direct interaction with SEA. On the other hand, the low polymerized PCs might inhibit the toxic activity without interaction with SEA. Goto et al. (2015) reported that apple PCs and their components suppressed IFN-γ secretion through activated T cells. In addition, it was demonstrated that oligomeric PCs interfered with glycolysis and inhibited the functions of activated T cells [22]. It was suggested that oligomeric PCs have an influence on the interaction of ligands and their receptors on the cell surface, including the surface of CD4+ T cells [23,24,25]. From these reports, we assume that oligomeric procyanidin may suppress T cell activation by interacting with their receptors. Therefore, it will be necessary to examine the interaction of oligomeric PCs with their receptors in CD4+ T cells. Superantigens can also cause toxic shock syndrome, a serious condition characterized by rashes, hypovolemic shock and respiratory distress syndrome [26]. Therefore, these proanthocyanidins may control the SEA-induced various symptoms mentioned above. Further studies on the inhibitory mechanisms of polyphenols on SEA cytotoxicity may be needed.

Based on the finding that low polymerized PCs inhibit the production of SEA, we investigated the relative expression levels of *sea* toxin encoding genes after treatment with low polymerized PCs. All low polymerized PCs significantly inhibited the transcription of SEA in *S. aureus* C-29 (Figure 7). These results suggest that although low polymerized PCs do not interact with SEA, they inhibit not only SEA toxin activity, but also the production of SEA. This is the first report to demonstrate that highly polymerized PCs interact with SEA and inhibit SEA induced toxin activity; low polymerized PCs inhibit production of SEA. The PCs in apples might be a useful tool for prevention of food poisoning. A new series of experiments using PCs not of apple origin are required to test the inhibitory effects on SEA toxin activity. It is thereby expected that mechanisms of a more detailed interaction between PCs and SEA will be clarified.

## 4. Materials and Methods

### 4.1. Bacterial Strains

The SEA-producing strain *S. aureus* C-29 was used through all the studies. The culture method of bacterial strains *S. aureus* C-29 has been described in detail previously [8].

### 4.2. Fractionation of AP Mixture

The separation and fractionation of the AP mixture was performed as reported by Yanagida et al. (2007) [27]. The AP mixture (Applephenon^®^, Asahi Food and Healthcare Co. Ltd., Tokyo, Japan) an apple polyphenol extract produced commercially from unripe apples) was separated by preparative HPLC using a TSKgel Amide-80 column (21.5 × 300 mm I.D., 10 μm, Tosoh Corporation, Tokyo, Japan) in hydrophilic interaction chromatography mode. Mobile phases A and B were mixtures of acetonitrile and water at volume ratios of (A) 90:10 and (B) 50:50, respectively. Lyophilized powder (100 mg) of methyl acetate extract of the AP mixture was dissolved in 2 mL of solvent A (final conc.: 50 mg/mL). The full volume of this sample solution was injected and eluted in accordance with a linear gradient program from 100% A (0% B) to 0% A (100% B) for 60 min at a flow rate of 12 mL/min. The absorbance of effluent was monitored using a diode array detector (200–400 nm), and the effluent was fractionated using a fraction collector. Two preparative HPLC runs were made, and the respective PC fractions were combined afterwards. In total, 200 mg of extract of the AP mixture was fractionated. The mg of each fraction was determined by lyophilization and following weight measurement. Furthermore, the oligomeric constituents of PCs in each collected fraction were analyzed by HPLC using a conventional size of TSKgel Amide-80 column (4.6 × 250 mm I.D., 5 μm) under the following conditions; mobile phases, A and B were mixtures of acetonitrile and water at volume ratios of (A) 90:10 and (B) 50:50, respectively; linear gradient program, 100% A (0% B) to 0% A (100% B) for 60 min; flow rate 1.0 mL/min; detection wavelength, 200–400 nm).

### 4.3. Interaction between PC Fractions and Cultured SEA-Producing Strain or Purified SEA

The concentration of the six obtained PC fractions (mono-, di-, tri-, tetra-, penta-, and greater than pentamer) was used in the minimum inhibitory concentration (MIC) values or less. The testing method for MIC was performed as previously described [8]. Each fraction was tested at a final concentration of 0.1, 0.25, 0.5, 1.0, 1.5, 3.0 mM diluted in dimethyl sulfoxide. The interaction between each fraction and SEA-producing strain *S. aureus* C-29 or highly purified SEA (greater than 95% purity; Toxin Technology, Sarasota, FL, USA; final concentration 100 ng/mL) was estimated as previously described [8,13]. The resultant supernatant was used for SDS-PAGE and Western blot analysis.

### 4.4. HPLC Analysis

In a second measurement of the interaction between each PC fraction and purified SEA, and HPLC analysis detection of each PC fraction was performed. Each PC fraction (final concentration of 1.5 mM) and purified SEA (final concentration of 100 ng/mL) were mixed and incubated at 37 °C for 24 h. After incubation, the reaction mixture was centrifuged at 6000× *g*, and the supernatant was passed through a 0.45-μm pore size membrane filter before injection. Each fraction was analyzed by HPLC (column, Agilent Zorbax SB-C18 (4.6 × 150 mm I.D., 1.8 μm, Agilent Technologies, Palo Alto, CA, USA); mobile phase 0.5% trifluoroacetic acid (TFA):acetonitrile (87:13); flow rate 0.6 mL/min; detection, 525 nm).

### 4.5. Biacore Analysis

Biomolecular analysis of the interaction of apple polyphenol (AP) with SEA was examined by SPR with a Biacore 2000 biosensor instrument (Pharmacia Biosensor, Uppsala, Sweden). Immobilization of SEA proteins on a sensor chip CM5 (GE Healthcare, Buckinghamshire, UK) was performed using an amine coupling kit according to the manufacturer’s manuals. For initial binding analysis, SEA was immobilized on separate channels on a CM5 chip, to 966.5 RU. AP was assayed over the immobilized SEA sensor surface at room temperature. AP (0, 3.75, 5.0, 12.5, 25, 37.5 and 50 μg/mL) in an HBS-EP running buffer (0.01 M HEPES pH 7.4, 0.15 M NaCl, 3 mM EDTA, 0.005% (*v*/*v*) Surfactant P20; GE Healthcare) were injected. Regeneration was performed by injecting a 10 mM Glycine-HCl solution at pH 3.0 (GE Healthcare) over the sensor chip surface. The resonance angle was measured in resonance units (RU).

### 4.6. Antibody Production against SEA Active Sites

There are four SEA functional regions (peptides) that are responsible for superantigenic and emetic activities on SEA structures [14]. According to the amino acid sequence of SEA reported previously, these four peptides (20 amino acid residues in length), corresponding to the fragment of the primary sequence of SEA, were synthesized. The binding affinities of AP to SEA toxin active sites were analyzed using four synthetic peptides equivalent to SEA toxin active sites, and rabbit antibodies to their corresponding peptides (A-2, A-3, A-6 and A-10). A-2, A-3, A-6 and A-10 peptides, corresponding to the regions 21–40, 35–50, 81–100, or 161–180 in SEA toxin active sites [14], were obtained from Sigma-Aldrich Corporation (St. Louis, MO, USA). The synthetic peptides are as follows: CALGNLKQIYYYNEKAKTEN (A-2), CKAKTENKESHDQFLQH (A-3), KGKKVDLYGAYYGYQC (A-6), CRYLQEKYNLYNSDVFDGKV (A-10). In A-2 (based on the amino acid sequence 22 to 40 of SEA), C was added to the N-terminal side, and T was deleted to decrease high hydrophobicity at the N-terminal side. A-3 (based on the amino acid sequence 35 to 50 of SEA) was added C to the N-terminal side. A-6 (based on the amino acid sequence 81 to 96 of SEA) terminates with a C, therefore no changes (additions/deletions) were necessary. In A-10 (based on the amino acid sequence 161 to 179 of SEA), C was added to the N-terminal side, and Q was deleted to the C-terminal side to maintain the charge of the R at the N-terminal side. The rabbit antibodies and their corresponding peptides were used at a ratio of 1:1000. SDS-PAGE and Western blot analysis were performed as previously described [8]. A protein setector Western blotting kit, BCIP/NBT System (KPL, Gaithersburg, MD, USA), was used to detect the SEA active site signals according to the manufacturer's instructions.

### 4.7. Digestive Tract Model

We estimated the interaction of samples with SEA using in vitro intragastric enteral models to test the action of samples on SEA within the digestive tract. In this study, we investigated the effects of pH, pepsin and pancreatin treatments on the interaction of AP to SEA. To examine the effects of pH (Mcilvaine buffer; pH 4.0, 6.0, 6.8 and 8.0) on the interaction of AP with SEA, AP (2.5 mg/mL) and SEA mixed in each pH buffer solution and incubated at 37 °C for 24 h. We also examined the effects of hydrolysis by gastrointestinal proteases. AP (2.5 mg/mL) and SEA mixture was adjusted to a pH of 2.4 using Mcilvaine buffer, followed by the addition of pepsin from porcine gastric mucosa (Sigma-Aldrich; final concentration 0.32%), and incubated at 37 °C for three hours. After three hours, the solution was adjusted from a pH of five to a pH of six with 10 M NaOH of inactivate pepsin, followed by the addition of pancreatin from porcine pancreas (Sigma-Aldrich; final concentration 0.25%) and bile powder (final concentration 0.4%), and incubated at 37 °C for 24 h. Because the pH values in small intestine are in the range of 5.0–6.5 and effective pH range of pancreatin is pH 4.75 to 7.0, pH was adjusted to pH 5.0 to 6.0. After 24 h, the reaction mixture was used for SDS-PAGE and Western blot analysis.

### 4.8. Isolation of Mouse Spleen Cells and SEA Activity Assay

The separation of mouse spleen cells from C57BL/6J female mice was conducted as previously described [21]. Inhibitory effects of samples on SEA activity was performed by an enzyme cleavage assay as reported by Rasooly et al. (2010), with some modifications [4]. Briefly, 45 µL of spleen cells (1 × 10^5^ cell/mL), 49 µL of Russ-10 medium, 5 µL of AP (final concentration 0.1 mg/mL) or each PC fraction (0.1 mM), and with or without 1 µL SEA (100 ng/µL) were mixed in 96-well plates and followed by incubation at 37 °C in a 5% CO_2_ incubator for 48 h. AP was a mixture containing from PC monomer to greater-than-pentamers and could not be showed in mM. The weight concentration (mg/mL) of each PC fraction in AP at 0.1 mM was 0.03 mg/mL (monomer), 0.06 mg/mL (dimer), 0.09 mg/mL (trimer), 0.12 mg/mL (tetramer), 0.14 mg/mL (pentamer) and 0.17 mg/mL (greater than pentamer). Cytotoxicity induced by SEA with or without samples was estimated with the MultiTox-Fluor Multiplex Cytotoxicity Assay (Promega Co., Madison, WI, USA) according to the manufacturer’s manuals. Animal experimental procedures were performed with the approval of the Institutional Animal Care and Use Committees of the University (Permit Number: 145058, 145074 and 165112; Date of approval: 5 May 2014, 30 March 2015 and 24 March 2016).

### 4.9. Cytokine Detection

Forty-five µL of spleen cells were placed in 96-well plates (1 × 10^5^ cell/mL) in 49 µL of Russ-10 medium and 5 µL of AP (final concentration 0.1 µg/mL) or each PC fraction (0.1 mM) was added with or without 1 µL SEA (100 ng/µL), followed by incubation at 37 °C in a 5% CO_2_ incubator for 72 h. The inhibitory effects of samples on IFN-γ production in spleen cell culture supernatants was estimated with the mouse IFN-γ ELISA kit (RayBio, Norcross, GA, USA) according to the manufacturer’s manuals.

### 4.10. RNA Isolation and Real-Time RT-PCR

RNA isolation and real time RT-PCR was performed as previously described [21]. Samples for RNA extraction were taken at two different times (three hour and 6.5-hour-old culture). Briefly, the *S. aureus* strain C-29 in one mL of BHI broth was incubated with or without AP (final concentration of 2.5 mg/mL) or PC fraction (final concentration 3.0 mM). RNA extraction was performed using a RiboPure-Bacteria kit (Ambion, Austin, TX, USA), according to the manufacturer’s instructions. The real-time PCR was performed using a SYBR Premix Ex Taq (Takara, Shiga, Japan) and a real time PCR system (Thermal Cycler Dice^®^ Real Time System Single; Takara). Each sample was normalized to 16S rRNA, triplicates were averaged, and relative mRNA levels were determined.

### 4.11. Statistical Analysis

The results were analyzed using a Student t test or one-way ANOVA, followed by the Dunnett’s test using Microsoft Excel 2013 (Microsoft, Redmond, WA, USA). The significance level was set at *p* < 0.05 or < 0.01 and all experiments were replicated at least three times, except HPLC analysis data of Figure 3 (two replicates). The Tukey–Kramer test was used to compare differences between groups (*p* < 0.05).
